# Co-infections of *Plasmodium knowlesi*, *P. falciparum,* and *P. vivax* among Humans and *Anopheles dirus* Mosquitoes, Southern Vietnam

**DOI:** 10.3201/eid1707.101551

**Published:** 2011-07

**Authors:** Ron P. Marchand, Richard Culleton, Yoshimasa Maeno, Nguyen Tuyen Quang, Shusuke Nakazawa

**Affiliations:** Author affiliations: Medical Committee Netherlands–Vietnam, Khanh Hoa Province, Vietnam (R.P. Marchand, N.T. Quang);; Institute of Tropical Medicine, Nagasaki University, Nagasaki, Japan (R. Culleton, S. Nakazawa);; Fujita Health University School of Medicine, Aichi, Japan (Y. Maeno).

**Keywords:** forest malaria, epidemiology, monkey malaria, zoonotic malaria, research

## Abstract

A single *Anopheles dirus* mosquito carrying sporozoites of *Plasmodium knowlesi, P. falciparum,* and *P. vivax* was recently discovered in Khanh Phu, southern Vietnam. Further sampling of humans and mosquitoes in this area during 2009–2010 showed *P. knowlesi* infections in 32 (26%) persons with malaria (n = 125) and in 31 (43%) sporozoite-positive *An. dirus* mosquitoes (n = 73). Co-infections of *P. knowlesi* and *P. vivax* were predominant in mosquitoes and humans, while single *P. knowlesi* infections were found only in mosquitoes. *P. knowlesi*–co-infected patients were largely asymptomatic and were concentrated among ethnic minority families who commonly spend nights in the forest. *P. knowlesi* carriers were significantly younger than those infected with other malaria parasite species. These results imply that even if human malaria could be eliminated, forests that harbor *An. dirus* mosquitoes and macaque monkeys will remain a reservoir for the zoonotic transmission of *P. knowlesi.*

## Abstract 2

TOC Summary: Forests harboring these mosquitoes may be a reservoir for transmission of *P. knowlesi*.

Concerted control measures have considerably reduced the prevalence of malaria in Vietnam, and the parasites that cause it are now mostly restricted to forested rural areas (1). Forest malaria poses a special challenge for control because the exophilic and early biting habits of the mosquito vector *Anopheles dirus* render conventional vector control methods such as indoor residual spraying and insecticide treated mosquito nets difficult to apply as well as ineffective (2–4). The possibility of zoonotic malaria in Southeast Asian forests, because of the transmission of *Plasmodium knowlesi* from monkeys to humans (5–10), may form an additional complication.

Since surveys began in 2002, the forest populations of *An. dirus* mosquitoes in Khanh Phu, south-central Vietnam, have shown consistently high sporozoite infection rates (1%–2% of the thousands of specimens collected and dissected annually for microscopic examination of salivary glands), raising the question of whether all the sporozoites detected belong to species capable of infecting humans. In 2008, evidence was found for the co-infection of *P. knowlesi*, *P. falciparum,* and *P. vivax* in the salivary glands of 1 mosquito among 17 that had been processed by PCR with malaria parasite species–specific primers (11). Here we report the results of the PCR analysis of 72 additional sporozoite–positive salivary glands of *An. dirus* mosquitoes from the forest in Khanh Phu and of 211 blood samples from the local human population.

## Study Population and Methods

### Study Area

Khanh Phu (12°14′N; 108°56′E) is a commune with ≈3,000 inhabitants, mainly of the Raglai ethnic minority, who live between the forested foothills on the east side of the Truong Son mountain range in south-central Vietnam (Khanh Hoa Province), an area where malaria was previously hyper- to holo-endemic (3). Since 1993, the Medical Committee Netherlands–Vietnam (a Dutch nongovernmental organization) has cooperated with the National Institute of Malariology, Parasitology and Entomology in Hanoi; the Institute of Malariology, Parasitology and Entomology in Qui Nhon; and the Malaria Control Centre of Khanh Hoa Province to set up and operate the Khanh Phu Malaria Field Research Unit to provide Vietnamese malaria researchers with the opportunity to study local malaria epidemiology and develop and test improved control methods. *An. dirus* species A is currently the only malaria-transmitting species of mosquitoes in Khanh Phu. *Anopheles minimus* mosquitoes, previously the major malaria vector in the region, disappeared from Khanh Phu after 1998, following the wide-scale use of insecticide-treated mosquito nets (3,12). While the average malaria prevalence in the human population has been greatly reduced (from >50% before 1998 to 2%–3% during 2003–2009), persons who sleep overnight in the forest still run a high risk for infection. Analysis of samples from infected persons by thin-smear microscopy showed that approximately two thirds were caused by *P. falciparum*, one third by *P. vivax,* and a very small number by *P. malariae* during the 2003–2009 study period. This malaria prevalence typically affects the poorest members of the local Raglai ethnic community, whose livelihood partly depends on excursions into the forest to collect products such as bamboo and rattan, or to cultivate their plots on the mountain slopes. All residences within the commune were mapped, and all persons were registered at the research station and assigned unique code numbers based on residential location and family relations. (All methods in this study that involved human participants in the field were certified as permitted standard procedures by the National Institute of Malariology, Parasitology and Entomology in Hanoi.)

### Mosquito Collection and Salivary Gland Examination

Mosquitoes were collected by human-baited landing catches at 4 collection sites in the forest and forest fringe areas near Nga Hai village in the southern part of Khanh Phu commune, from January 2008 through February 2010. Mosquito collectors were adult men of the Raglai ethnic group. They were intensively screened for malaria and promptly treated with artemisinin combination therapy if infected. The collectors worked in teams of 2 over the whole night, 1 person collected from 6:00 pm to 12:00 am and the other from 12:01 am to 6:00 pm. The monthly collection effort ranged between 40 and 60 person-nights per month, a total 1,285 person-nights over the 26-month period.

*Anopheles* species were determined on the basis of morphologic features (13). All *An. dirus* group mosquitoes were assumed to be *An. dirus* species A on the basis of previous accurate identifications and the known distribution of this species (14,15). Female anopheline mosquitoes were dissected for salivary glands, midgets, and ovaries, and these were examined by microscopy for the presence of sporozoites, oocysts, and parity, respectively. Sporozoite-infected glands were applied to filter paper and dried in an ambient atmosphere before storage in closed vials at 4°C–6°C.

### Collection of Human Blood Samples

Blood samples were collected by 2 methods. First, to detect as many parasite-infected persons as possible, from March 8, 2009, through February 28, 2010, blood was collected by targeted active case detection from persons who had frequently worked in the forest or had reported fever. A total of 549 blood samples from 305 persons from 156 families were collected; 183 persons were sampled once, and no one was sampled >8 times; the sex ratio had a male bias (64% male). Sixty-nine of the 121 blood samples that were positive by microscopy, and an additional randomly selected 105 of the negative samples were processed by PCR to determine *Plasmodium* species. Second, from March 11, 2010, through March 30, 2010, a cross-sectional survey was undertaken in the 2 villages nearest to the mosquito collection sites. A single blood sample was taken from each of 624 residents, irrespective of symptoms or work place. One hundred and thirty-five persons in this sample had previously given samples as part of the targeted active case detection (ACD) survey; 489 had not given samples previously. From the 49 blood samples found positive by microscopy, 37 were randomly chosen for PCR processing.

Sixty-eight percent of all blood slides in both samples were from persons in the 2 southernmost villages of Khanh Phu (Nga Hai and Da Trai). This represents an area where ≈1,100 persons are living, spread over an area of 1.7 km^2^ at 12°12.5′N and 108°55.5′E. All their houses are located <1 km from the nearest forest and 1–3 km away from the mosquito collection sites. *An. dirus* mosquitoes were rarely caught in these villages.

Blood was collected by finger-prick; thick and thin blood films were made for diagnosis, and blood was applied to filter paper for downstream molecular analyses. All adult volunteers provided informed consent and for children, consent was obtained from close relatives. All persons found to be infected with parasites (as diagnosed by microscopy) received treatment, according to the policy of the Vietnam Ministry of Health.

### DNA Extraction and Parasite Species Identification by PCR

DNA was extracted from dried blood samples on filter paper and from sporozoite-infected glands, and subsequent malaria parasite species identification by PCR that targeted the 18S rRNA gene was carried out as previously described (11,16). Briefly, DNA was extracted by using the QIAamp DNA micro kit (QIAGEN, Tokyo, Japan). Extracted DNA samples were stored at –20°C until use. *Plasmodium* species–specific nested PCR assays to detect and identify human malaria parasites were performed as described (17). For detection of the *P. knowlesi* 18S rRNA gene, the primers Pmk8 and Pmk9 were used (5). Because this primer set can occasionally cross-react with *P. vivax* DNA and produce false-positive results (18), samples were also subject to a further PCR targeting of the *P. knowlesi* circumsporozoite protein (CSP) gene as described (8). PCR products were separated by electrophoresis on 1.5% agarose gels and stained with ethidium bromide. Primer sequences for the gene of human *Plasmodium* species (17), 18S rRNA of *P. knowlesi* (5), and the CSP gene of *P. knowlesi* (8) were as previously described. *P. knowlesi* H strain (American Type Culture Collection no. 30158) (kindly donated by Satoru Kawai, Dokkyou University, Japan) was used as a positive control. We verified that no cross-reaction occurred between the primer sets used to amplify *P. vivax* CSP and *P. knowlesi* CSP by using DNA extracted from single infections of both species (data not shown).

### Statistical Analysis

Student 1- or 2-tailed t tests were used for comparing means. Chi-square tests for association and confidence limits were calculated with Microsoft Excel (Microsoft Corp., Redmond, WA, USA).

## Results

### *Plasmodium* Infections of *An. dirus* Mosquitoes

From January 1, 2008, through February 22, 2010, a total of 6,834 female anopheline mosquitoes were captured, of which 83.2% belonged to *An. dirus s.s*., and 8.6% to *An. maculatus*, 5.4% to *An. peditaeniatus*; the remaining 2.8% were divided between a further 11 anopheline species in very small numbers. Of the 8,331 *An. maculatus* mosquitoes dissected in Khanh Phu over >15 years, only 1 mosquito was ever found to be infected with sporozoites. None of the other *Anopheles* species, with the exception of *An. dirus*, were ever found to be infected with sporozoites. Thus *An. dirus* mosquitoes are regarded as the only malaria vectors in the Khanh Phu forest.

*An. dirus* mosquitoes were found during every month of sampling with an average human-biting density of 4.4 bites/person-night (range 0.3–17.4; Table A1). As in previous years in Khanh Phu (3), these mosquitoes usually reached peak densities in the dry season (February–April). Numbers were lowest during the hottest part of the year (May–August), climbed to a second peak with the onset of the rains during September–November, and usually decline again following heavy rains during October–December. The monthly average parous rate fluctuated between 65% and 90% (average 77%).

Sporozoites were detected by microscopic screening in 89 (1.57%) of 5,663 dissected *An. dirus* mosquitoes, and oocysts were observed on 0.94% of the midguts. The sporozoite rate showed some seasonal fluctuations, but these did not clearly correlate with the fluctuations in biting density. Multiplying the sporozoite rate by the human-biting rate gave an average annual entomological inoculation rate of 25.4 infective bites per person per year. This is comparable with the average rate estimated during the previous 8 years in the forest of Khanh Phu (22 infective bites per person per year, unpub. data). However, both the sporozoite rate (4.6%) and human-biting density (12 bites/person-night) reached a maximum in the last 2 months of the survey (January and February 2010). This finding implies that persons who slept unprotected in the forest during this period would have had a >50% chance of being bitten by an infected *An. dirus* mosquito during any 1 night.

**Figure 1:**
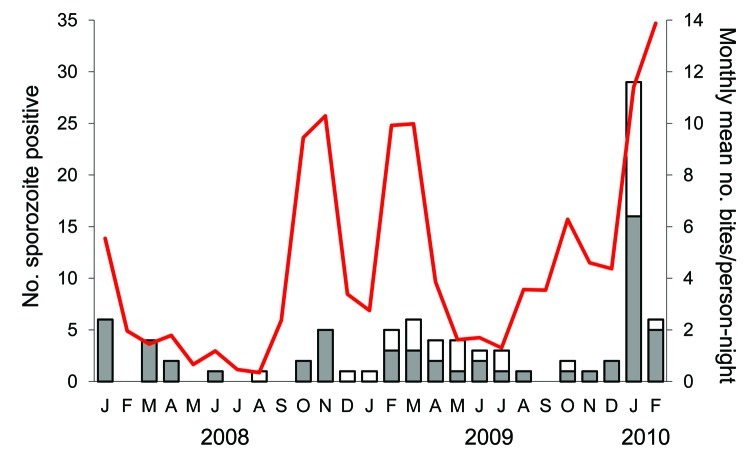
Results and interpretation of the PCR analyses of sporozoite-positive salivary glands of *Anopheles dirus* mosquitoes in Khanh Phu forest, Vietnam. CSP, circumsporozoite protein; ssu, small subunit; shaded cells, PCR products present; F, *Plasmodium falciparum*; V, *P. vivax*; K, *P. knowlesi*; M, *P. malariae*; ?, unknown.

Of the 86 sporozoite-positive mosquitoes, 73 underwent PCR analysis for malaria parasite detection, of which 72 were successfully assayed. Thirty-one (43%) of these 72 salivary glands were PCR positive for *P. knowlesi csp* and for *P. knowlesi* 18S rRNA. Five additional specimens were positive for *P. knowlesi* 18S rRNA but negative for *P. knowlesi* CSP and were therefore regarded as *P. knowlesi* negative (Figure 1). One specimen was only positive for *P. knowlesi* CSP and not by any other test, and therefore the species could not be confidently determined. The frequencies of *P. falciparum*, *P. vivax,* and *P. malariae* were: 50%, 50%, and 6%, respectively. In 22 (71%) of 31 glands, *P. knowlesi* was found as a co-infection with one of these other species. The combination *P. knowlesi* + *P. vivax* (in 14 glands) was far more common than *P. knowlesi* + *P. falciparum* (1 case). The combination of *P. knowlesi* + *P. vivax* + *P. falciparum* was, however, also quite common (7 glands).

**Figure 2:**
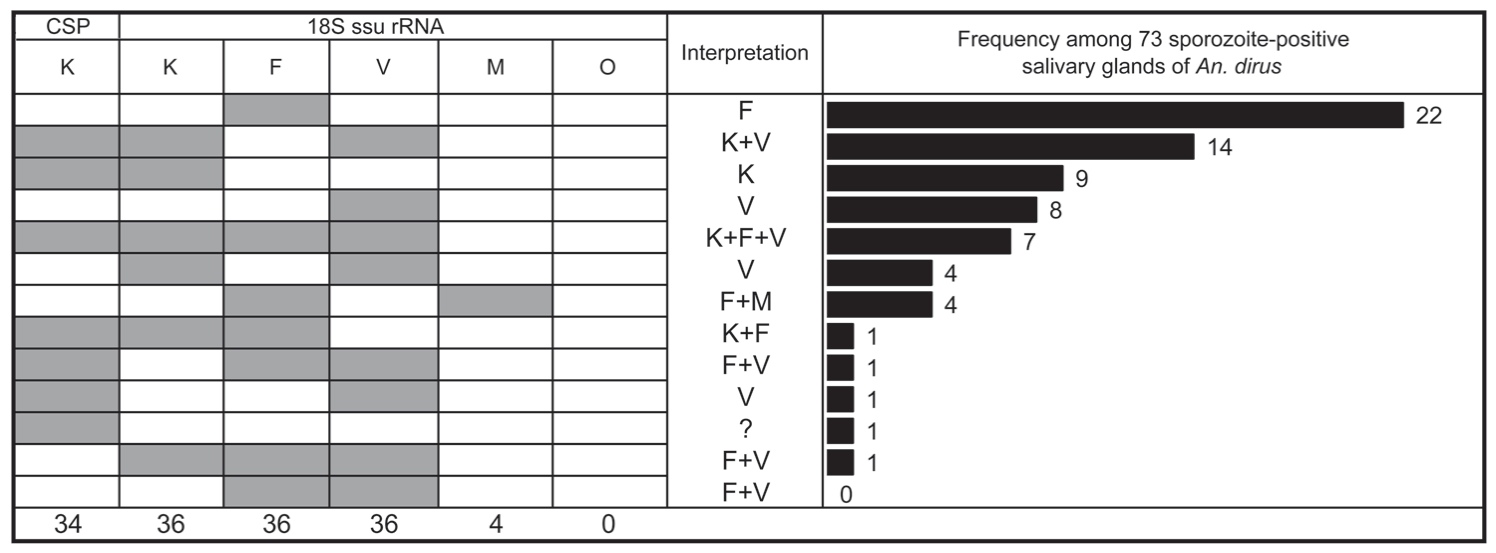
Dynamics of biting density and sporozoite positive salivary glands (including *Plasmodium knowlesi)* of *Anopheles dirus* mosquitoes in Khanh Phu forest, Vietnam. The solid red line connects the points of mean monthly *An. dirus* density (bites/person-night). Bars indicate the monthly number of sporozoite-positive salivary glands: white, *P. knowlesi*; gray, other species. The first mosquito found with *P. knowlesi* in August 2008 was described in Nakazawa et al. (11).

The frequency of *P. knowlesi*–positive mosquitoes did not significantly differ between the 4 collections sites, and *P. knowlesi* was detected during 8 months of the past year, except in August, September, November, and December when sporozoite-infected mosquitoes were few (Figure 2). However, the highest number of sporozoite-infected mosquitoes and 13 of the 31 *P. knowlesi* infections were found in January 2010.

### *Plasmodium* Infections of Humans

Of the 549 blood samples collected by targeted ACD from March 2009 through February 2010, 121 (22.0%) were parasite positive by microscopy, including 94 (17.1%) that were positive for *P. falciparum*. The cross-sectional survey conducted in March 2010 showed a parasite prevalence of 7.9% (10.5% in male residents and 5.2% in female residents; χ^2^ p = 0.013). The *P. falciparum* prevalence was 5.1% (7.3% in male residents and 2.9% in female residents; χ^2^ p = 0.012).

The Table shows the results of parasite species identification by microscopy and PCR for both the targeted ACD and cross-sectional surveys. Both samples had a male bias. In the targeted ACD sample, this result reflected the skewed sex ratio in the total 549 blood samples. Among the 624 persons sampled during the cross-sectional survey the sex ratio was 50/50, but because infections were more common in men, the random subsample of 37 for PCR analysis of the 49 positive results by microscopy was also male biased. The mean age of women and men was similar, and the difference in mean age between findings of the targeted ACD and those of the cross-sectional survey was not significant (*t* test p>0.05). In the targeted ACD sample, 19 (18%) of 105 samples that were negative by microscopic examination were parasite positive by PCR. Five patients with positive results in the ACD were shown by PCR to have *P. malariae* as a co-infection, and 1 patient in the cross-sectional survey had a *P. malariae* single infection, all of which were not seen by microscopy. We were unable to determine the parasite densities of *P. knowlesi* in our samples because it was not possible to discriminate between parasite species in co-infections at low parasite densities.

**Table:** Frequencies of malaria parasite species determined by microscopy and PCR in 2 samples of human blood from residents of Khanh Phu commune, Vietnam, 2009–2010*

Test categories	Active case detection†				Cross-sectional survey‡		
	Men	Women	Total		Men	Women	Total
No. tested	110	64	174		25	12	37
Patient mean age, y	25.7	25.1	25.5		20.7	20.0	20.4
By microscopy determination							
Total no. parasite positive	47 (43)	22 (34)	69 (40)		25 (100)	12 (100)	37 (100)
No. *Plasmodium falciparum* positive	38 (35)	15 (23)	53 (30)		16 (64)	4 (33)	20 (54)
No. *P. vivax* positive	14 (13)	8 (13)	22 (13)		13 (52)	8 (67)	21 (57)
By PCR determination							
Total no. parasite positive	60 (55)	28 (44)	88 (51)		25 (100)	12 (100)	37 (100)
No. *P. falciparum* positive	49 (45)	19 (30)	68 (39)		21 (84)	6 (50)	27 (73)
No. *P. vivax* positive	28 (25)	14 (22)	42 (24)		18 (72)	9 (75)	27 (73)
No. *P. knowlesi* positive	8 (7)	5 (8)	13 (7)		12 (48)	7 (58)	19 (51)
No. *P. malariae* positive	1	4	5		1	0	1
No. *P. ovale* positive	0	0	0		0	0	0
*Values are no. (%) except as indicated. All percentages are calculated over the total no. samples tested in each column. †The active case detection sample consisted of 174 blood samples of 549, including 69 of 121 found positive by microscopy. ‡In the cross-sectional survey, blood was taken from 624 persons, 49 of which were found positive by microscopy. A random selection of 37 blood samples of these 49 positive samples was analyzed by PCR.							

A total of 32 blood samples were found to be infected with *P. knowlesi* on the basis of simultaneous positivity in the *P. knowlesi* 18S rRNA and *P. knowlesi* *csp* PCR tests: 19 (51%) of 37 malaria-infected persons in the cross-sectional survey, and 13 (15%) of 88 PCR-positive cases collected by targeted ACD. Four additional specimens in the ACD sample were positive for *P. knowlesi* 18S rRNA but negative for *P. knowlesi* *csp*, all in co-infections with other parasite species, most commonly *P. vivax*. Human blood samples positive for *P. knowlesi* *csp* and negative for *P. knowlesi* 18S rRNA were not found.

*P. knowlesi* infection was detected by targeted ACD in 8 of 12 months. On the basis of the PCR results of the samples from the ACD survey, the annual *P. knowlesi* incidence can be estimated at 10/1,000 person-years (95% confidence limit 4%–17%). The results from the cross-sectional survey imply a peak *P. knowlesi* prevalence in March 2010 among the population of the southern villages of 12.6% (95% confidence limit 6.3%–21.1%). The large increase in *P. knowlesi* co-infections in humans in March 2010 correlates with the increased frequency of this parasite in the mosquitoes in the preceding months.

Twenty-eight of the 32 *P. knowlesi* infections were found in persons of Raglai ethnicity, the dominant group in the study area. However, 2 of the 5 persons of Trinh ethnicity and 2 of the 7 persons of Kinh ethnicity sampled also had a *P. knowlesi* co-infection; all had asymptomatic cases detected during the cross-sectional survey.

**Figure 3:**
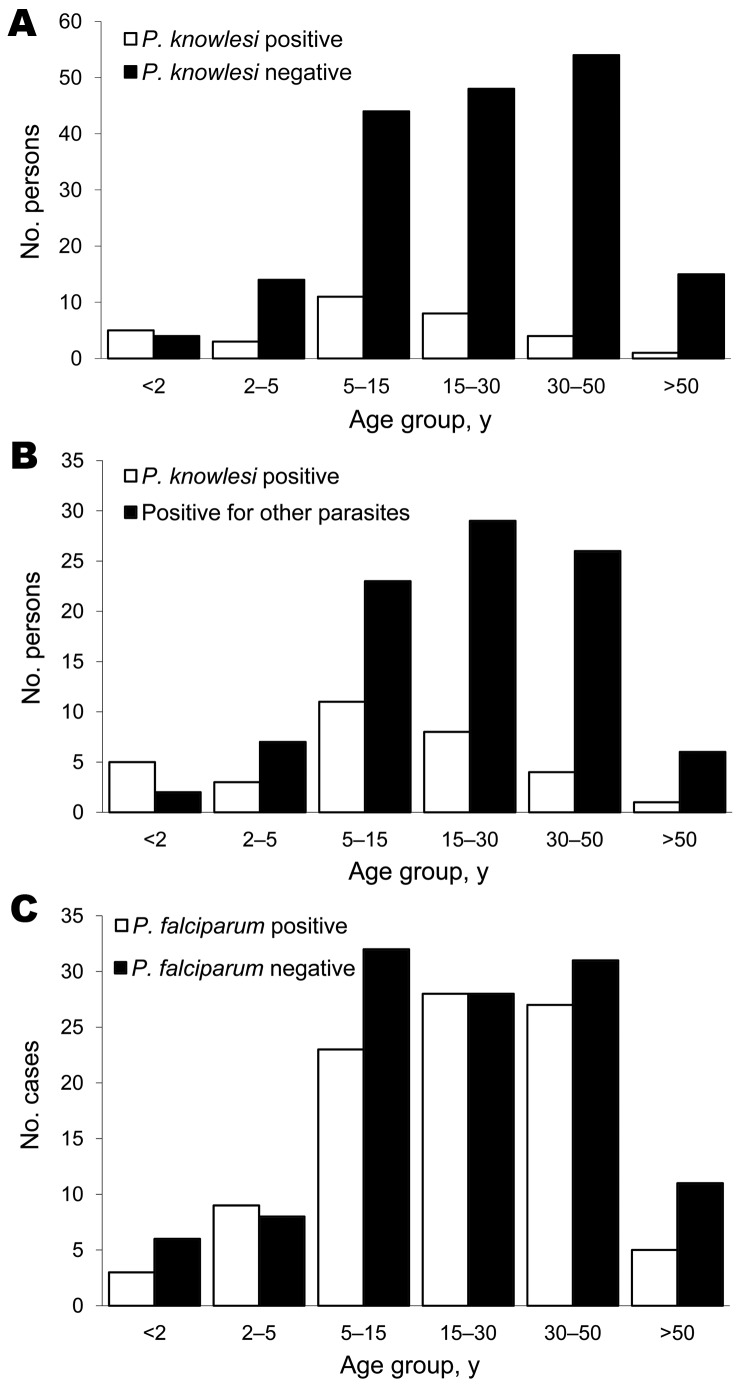
Age analysis of persons tested for *Plasmodium knowlesi* infection, Khanh Phu, Vietnam. A) Age groups of *P. knowlesi–*positive persons (n = 32; mean age 15.8 y) compared with *P. knowlesi*–negative persons (n = 179; mean age 26.2 y); p = 0.0004 (significant) by 2-tailed *t* test with unequal variance. B) Age groups of *P. knowlesi–*positive persons compared with ages of those positive for other parasites (n = 93; mean age 24.5 y); p = 0.004 (significant) by 2 tailed *t* test with unequal variance. C) Age groups of *P. falciparum*–positive persons (n = 95; mean age 23.4 y) compared with *P. falciparum*–negative persons (n = 116; mean age 25.6 y); p = 036 (not significant) by 2-tailed *t* test with unequal variance.

The average age of persons with *P. knowlesi* co-infections was 15.0 years in the targeted ACD survey and 16.9 years in the cross-sectional survey; both ages were significantly lower than the ages of *P. knowlesi–*infected persons in both samples (24.8 and 26.2 years, respectively; 2-tailed *t* test p<0.001; Figure 3, panel A) and those of all other positive case-patients (24.8 and 24.1 years, respectively; 2-tailed *t* test p< 0.01; Figure 3, panel B). *P. falciparum*–infected persons had a mean age of 23.4 years, which did not differ significantly from the mean age of 25.6 years for *P. falciparum*–negative persons (2-tailed *t* test p>0.3; Figure 3, panel C).

Six (19%) of the 32 *P. knowlesi* co-infected persons had fever. This finding was not significantly different from the 10% with fever among 86 uninfected persons in the PCR-analyzed sample (χ^2^ p = 0.23). Only persons with single *P. falciparum* infections were significantly more often febrile (30%) than uninfected persons (χ^2^ p = 0.003).

**Figure 4:**
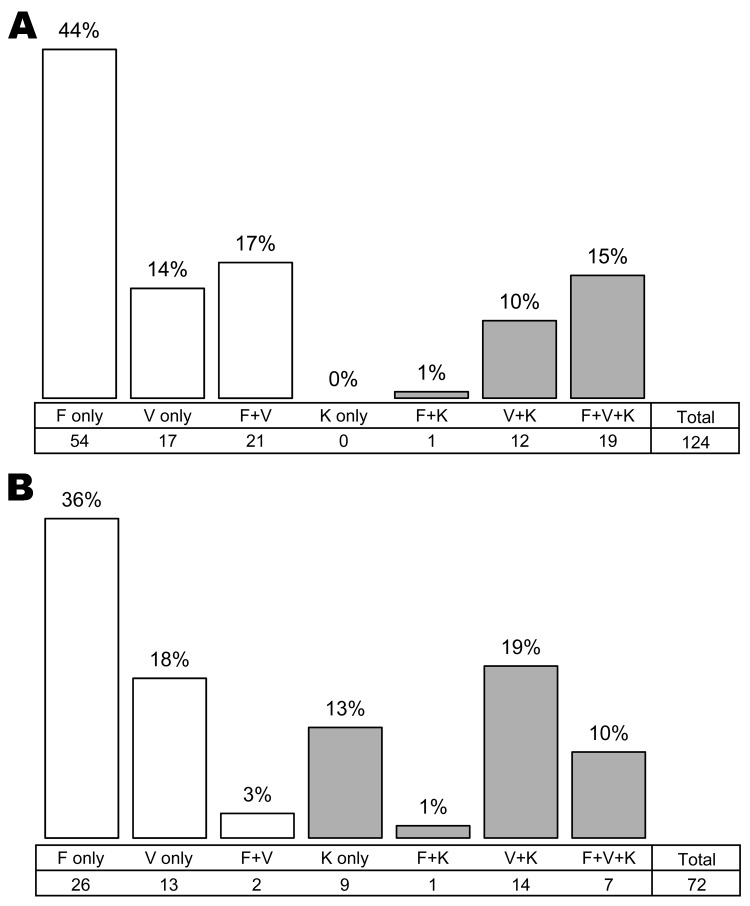
Frequency of single and co-infections among 124 human blood samples (A) and 73 mosquito salivary glands (B) positive for *Plasmodium* spp. infection by PCR. *P. malariae* was discarded. Gray bars indicate *P. knowlesi* infection or co-infection. F, *P. falciparum*; V, *P. vivax*; K, *P. knowlesi*.

In the human blood samples, *P. knowlesi* was only ever found in a co-infection, almost always with *P. vivax* and often with *P. falciparum* in addition. The lack of the *P. knowlesi* and *P. falciparum* without *P. vivax* combination (only 1 case each in the mosquito and human samples; Figure 4, panels A and B) contributed most strongly to the significance of the nonrandom distribution (χ^2^ p<0.001). *P. knowlesi* infection was equally frequent among PCR-positive women and men, whereas *P. falciparum* was significantly more frequent among the PCR-positive men (Table).

## Conclusions

*P. knowlesi* occurs frequently in humans in Khanh Phu as well as in the *An. dirus* mosquito population in nearby forests. This finding, in combination with the increasing number of reports of *P. knowlesi* infections from Thailand (6) and Myanmar (10), in similar forested environments, highlights the wide range of this pathogen in humans in Southeast Asia.

Our results provide additional information about vector bionomics and the clinical manifestation and epidemiology of *P. knowlesi* in a native population. *P. knowlesi* occurs in high frequencies as co-infections in *An. dirus* species A mosquitoes, which is the only human malaria vector in this area (3). Other anopheline species thus far proven to transmit *P. knowlesi* between macaque monkeys and humans in Southeast Asia, *A. cracens* and *A. latens* (8,19), belong, together with *An. dirus* species, to the Leucosphyrus group. Due to the widespread distribution of several *An. dirus* species in Southeast Asia (14) a prominent role of *An. dirus* sensu lato in sylvatic and zoonotic malaria transmission seems likely.

In our study, *P. knowlesi* infections in humans were always associated with infections of other *Plasmodium* species. This finding was in contrast to infections in mosquitoes, in which *P. knowlesi* single infections were relatively common. Furthermore, the combination of *P. falciparum* + *P. knowlesi* was far less common in both humans and mosquitoes than would be expected by chance. This may suggest a degree of interaction between these species that precludes the establishment of co-infections. Such nonrandom association was, however, not found in studies from Thailand, Malaysia, and Myanmar (6,8,10).

Even though known to be potentially dangerous for humans (20–22), the *P. knowlesi* co-infections described here did not lead to severe disease or fever and were concentrated in a group of ethnic minority families who often work in or near the forest, confirming a report from a neighboring area (23). The lack of symptoms, high sensitivity of the PCR method, and the fact that neither *P. knowlesi* nor *P. malariae* (which is often mistaken for *P. knowlesi*) was observed by microscopy suggest that the human *P. knowlesi* co-infections in this area cause very low levels of parasitemia. *P. knowlesi*–infected persons were on average significantly younger (15.8 years) than those infected with other species (23.9 years), which suggests that natural immunity is more easily acquired against *P. knowlesi* than against *P. falciparum* and *P. vivax*.

Macaques are common in the forests of Khanh Phu and are likely to be bitten by the same *An. dirus* population that bites humans. Whether these monkeys harbor a zoonotic malaria reservoir of *P. knowlesi* or if the parasite is also transmitted from person to person is currently unknown and requires further investigation. In this study, only *P. knowlesi, P. falciparum, P. vivax,* and *P. malariae* were investigated. Therefore whether *P. knowlesi* is the only malaria parasite in monkeys transmitted by *An. dirus* mosquitoes in this area is unknown.

Recent discoveries have demonstrated the relative ease and frequency at which malaria parasites may have switched between hosts (24–29). The factors that influence the probability of such host switching are likely to be many and varied, but the presence of a mosquito vector that brings the parasites in contact with different hosts must be a key precondition. *An. dirus* species A appears to fit this role.

### Epidemiologic and Public Health Implications

These findings may fundamentally change the perspectives for the control of forest malaria. Previously, forest malaria may have been considered manageable because the parasite reservoir in the forest may be reduced through intensive case detection and treatment of human communities living in or near the forests. The likely presence of a nonhuman reservoir of *P. knowlesi* (and possibly other parasites) in monkeys and of a mosquito vector that intensively inoculates parasites among monkeys and humans reinforces the need to find methods of vector control or biting prevention that can be applied to *An. dirus* mosquitoes.

## Appendix

**Table A1:** Results of the collection, dissections and PCR processing of *Anopheles dirus* mosquitoes caught by human landing catch in the forest of Khanh Phu, Vietnam, 2009–2010

	*An. dirus* collections			Midgut		Ovaries		Salivary glands			PCR tested	
Date, y, mo	No. caught	No. nights	Biting density*	No. dissected	No. oocysts.	No. parous	No. not parous.	No. dissected	No. sporozoites.	EIR†	No. PCR	No. P.k‡
2008												
Jan	194	35	5.5	146	1	161	33	194	6	0.17	2	–
Feb	47	26	1.8	27	–	42	5	47	–	0.00	–	–
Mar	87	60	1.5	41	1	58	27	87	4	0.07	–	–
Apr	107	60	1.8	45	1	74	33	107	2	0.03	2	–
May	39	59	0.7	21	–	30	9	39	–	0.00	–	–
Jun	71	60	1.2	42	–	54	17	71	1	0.02	1	–
Jul	28	60	0.5	16	–	24	4	28	–	0.00	–	–
Aug	20	58	0.3	7	–	17	3	20	1	0.02	1	1§
Sep	135	57	2.4	56	–	98	36	134	–	0.00	–	–
Oct	567	59	9.6	293	1	456	111	567	2	0.03	–	–
Nov	607	59	10.3	335	1	428	179	607	5	0.08	3	
Dec	203	60	3.4	116	2	156	47	203	1	0.02	1	1
2009												
Jan	88	32	2.8	39	–	79	9	88	1	0.03	1	1
Feb	387	40	9.7	198	2	307	78	385	5	0.13	4	2
Mar	499	50	10.0	282	1	380	119	499	6	0.12	6	3
Apr	193	50	3.9	106	2	149	44	193	4	0.08	4	2
May	81	47	1.7	35	–	70	11	81	4	0.09	4	3
Jun	85	50	1.7	37	–	63	22	85	3	0.06	3	1
Jul	65	49	1.3	45	2	56	8	64	3	0.06	3	2
Aug	178	49	3.6	89	–	142	36	178	1	0.02	1	–
Sep	220	50	4.4	131	–	158	50	208		0.00	–	–
Oct	364	50	7.3	209	1	272	93	365	2	0.04	2	1
Nov	278	50	5.6	175	–	184	100	284	1	0.02	1	–
Dec	380	50	7.6	215	4	290	81	371	2	0.04	2	–
2010												
Jan	559	49	11.4	345	9	453	108	561	29	0.59	29	13
Feb	222	16	13.9	132	2	153	44	197	6	0.42	3	1
Totals over 26 mo	5,704	1,285	4.44	3,183	30	4,354	1,307	5,663	89	0.07	73	31
												
			Averages:	% oocysts		% parous		% sporozoites		Annual EIR¶	% P.k positive#	
				0.94		76.9		1.57		25.46	42	

**Biting density = monthly average no. of bites by *An. dirus/*person-night; –, not found. †EIR, entomologic inoculation rate expressed as the monthly average no. of sporozoite-infected bites/person-night. ‡P.k, no. of mosquito salivary glands that were positive for *Plasmodium knowlesi*. §The analysis of the first mosquito found with *P. knowlesi* in August 2008 was described in Nakazawa et al. (11). ¶Annual EIR calculated from the average 0.07 infective bites/person-night over the whole period of 26 mo, then multiplied by 365. #The percentage of *P. knowlesi*–positive salivary glands among the sporozoite-positive salivary glands that underwent PCR.

## References

[1] Viet Nam National Institute of Malariology. Parasitology and Entomology (NIMPE). Annual reports of the National Malaria Control Program in Vietnam: 2003–2008. Hanoi: The Institute; 2008.

[2] Erhart A, Thang ND, Hung NQ, Toi LV, Hung LX, Tuy TQ, Forest malaria in Vietnam: a challenge for control. Am J Trop Med Hyg. 2004;70:110–8. 14993619

[3] Marchand RP. The Khanh Phu malaria research project: an overview 1994–2004. 2005. [cited 2010 Sep 4]. http://www.mcnv.nl/uploads/media/Malaria_overview_2005_18.pdf

[4] Erhart A, Ngo DT, Phan VK, Ta TT, Van Overmeir C, Speybroeck N, Epidemiology of forest malaria in central Vietnam: a large scale cross-sectional survey. Malar J. 2005;4:58. 10.1186/1475-2875-4-58 16336671 PMC1325238

[5] Singh B, Kim Sung L, Matusop A, Radhakrishnan A, Shamsul SS, Cox-Singh J, A large focus of naturally acquired *Plasmodium knowlesi* infections in human beings. Lancet. 2004;363:1017–24. 10.1016/S0140-6736(04)15836-4 15051281

[6] Putaporntip C, Hongsrimuang T, Seethamchai S, Kobasa T, Limkittikul K, Cui L, Differential prevalence of *Plasmodium* infections and cryptic *Plasmodium knowlesi* malaria in humans in Thailand. J Infect Dis. 2009;199:1143–50. 10.1086/597414 19284284 PMC8817623

[7] Luchavez J, Espino F, Curameng P, Espina R, Bell D, Chiodini P, Human infections with *Plasmodium knowlesi*, the Philippines. Emerg Infect Dis. 2008;14:811–3. 10.3201/eid1405.071407 18439369 PMC2600254

[8] Vythilingam I, Noorazian YM, Huat TC, Jiram AI, Yusri YM, Azahari AH, *Plasmodium knowlesi* in humans, macaques and mosquitoes in peninsular Malaysia. Parasit Vectors. 2008;1:26. 10.1186/1756-3305-1-26 18710577 PMC2531168

[9] Cox-Singh J, Singh B. Knowlesi malaria: newly emergent and of public health importance? Trends Parasitol. 2008;24:406–10. 10.1016/j.pt.2008.06.001 18678527 PMC2843823

[10] Jiang N, Chang Q, Sun X, Lu H, Yin J, Zhang Z, Co-infections with *Plasmodium knowlesi* and other malaria parasites, Myanmar. Emerg Infect Dis. 2010;16:1476–8. 10.3201/eid1609.100339 20735938 PMC3294981

[11] Nakazawa S, Marchand RP, Quang NT, Culleton R, Manh ND, Maeno Y. *Anopheles dirus* co-infection with human and monkey malaria parasites in Vietnam. Int J Parasitol. 2009;39:1533–7. 10.1016/j.ijpara.2009.08.005 19703460

[12] Garros C, Marchand RP, Quang NT, Hai NS, Manguin S. First record of *Anopheles minimus* C and significant decrease of *An. minimus* A in central Vietnam. J Am Mosq Control Assoc. 2005;21:139–43. 10.2987/8756-971X(2005)21[139:FROAMC]2.0.CO;2 16033115

[13] Vietnam National Institute of Malariology. Parasitology and Entomology. Identification key for Anophelinae in Vietnam. Hanoi (Vietnam): Medical Publishing House; 2008.

[14] Trung HD, Van Bortel W, Sochantha T, Keokenchanh K, Quang NT, Cong LD, Malaria transmission and major malaria vectors in different geographical areas of Southeast Asia. Trop Med Int Health. 2004;9:230–7. 10.1046/j.1365-3156.2003.01179.x 15040560

[15] Obsomer V, Defourny P, Coosemans M. The *Anopheles dirus* complex: spatial distribution and environmental drivers. Malar J. 2007;6:26. 10.1186/1475-2875-6-26 17341297 PMC1838916

[16] Maeno Y, Nakazawa S, Dao le D, Yamamoto N, Giang ND, Van Hanh T, . A dried blood sample on filter paper is suitable for detecting *Plasmodium falciparum* gametocytes by reverse transcription polymerase chain reaction. Acta Trop. 2008;107:121–7. 10.1016/j.actatropica.2008.05.001 18554563

[17] Singh B, Bobogare A, Cox-Singh J, Snounou G, Abdullah MS, Rahman HA. A genus- and species-specific nested polymerase chain reaction malaria detection assay for epidemiologic studies. Am J Trop Med Hyg. 1999;60:687–92. 10348249 10.4269/ajtmh.1999.60.687

[18] Imwong M, Tanomsing N, Pukrittayakamee S, Day NP, White NJ, Snounou G. Spurious amplification of a *Plasmodium vivax* small-subunit RNA gene by use of primers currently used to detect *P. knowlesi.* J Clin Microbiol. 2009;47:4173–5. 10.1128/JCM.00811-09 19812279 PMC2786678

[19] Tan CH, Vythilingam I, Matusop A, Chan ST, Singh B. Bionomics of *Anopheles latens* in Kapit, Sarawak, Malaysian Borneo in relation to the transmission of zoonotic simian malaria parasite *Plasmodium knowlesi.* Malar J. 2008;7:52. 10.1186/1475-2875-7-52 18377652 PMC2292735

[20] Jongwutiwes S, Putaporntip C, Iwasaki T, Sata T, Kanbara H. Naturally acquired *Plasmodium knowlesi* malaria in human, Thailand. Emerg Infect Dis. 2004;10:2211–3. 15663864 10.3201/eid1012.040293 PMC3323387

[21] Daneshvar C, Davis TME, Cox-Singh J, Rafe’ee MZ, Zakaria SK, Divis PCS, Clinical and laboratory features of human *Plasmodium knowlesi* infection. Clin Infect Dis. 2009;49:852–60. 10.1086/605439 19635025 PMC2843824

[22] Cox-Singh J, Hiu J, Lucas SB, Divis PC, Zulkarnaen M, Chandran P, Severe malaria—a case of fatal *Plasmodium knowlesi* infection with post-mortem findings: a case report. Malar J. 2010;9:10. 10.1186/1475-2875-9-10 20064229 PMC2818646

[23] Van den Eede P, Van HN, Van Overmeir C, Vythilingam I, Duc TN, Hung LX, Human *Plasmodium knowlesi* infections in young children in central Vietnam. Malar J. 2009;8:249. 10.1186/1475-2875-8-249 19878553 PMC2773789

[24] Escalante AA, Cornejo OE, Freeland DE, Poe AC, Durrego E, Collins WE, A monkey's tale: the origin of *Plasmodium vivax* as a human malaria parasite. Proc Natl Acad Sci U S A. 2005;102:1980–5. 10.1073/pnas.0409652102 15684081 PMC548581

[25] Hayakawa T, Culleton R, Otani H, Horii T, Tanabe K. Big bang in the evolution of extant malaria parasites. Mol Biol Evol. 2008;25:2233–9. 10.1093/molbev/msn171 18687771

[26] Duarte AM, Malafronte RS, Cerutti C Jr, Curado I, de Paiva BR, Maeda AY, Natural *Plasmodium* infections in Brazilian wild monkeys: reservoirs for human infections? Acta Trop. 2008;107:179–85. 10.1016/j.actatropica.2008.05.020 18620330

[27] Prugnolle F, Durand P, Neel C, Ollomo B, Ayala FJ, Arnathau C, African great apes are natural hosts of multiple related malaria species, including *Plasmodium falciparum.* Proc Natl Acad Sci U S A. 2010;107:1458–63. 10.1073/pnas.0914440107 20133889 PMC2824423

[28] Garamszegi LZ. Patterns of co-speciation and host switching in primate malaria parasites. Malar J. 2009;8:110. 10.1186/1475-2875-8-110 19463162 PMC2689253

[29] Liu W, Li Y, Learn GH, Rudicell RS, Robertson JD, Keele BF, Origin of the human malaria parasite *Plasmodium falciparum* in gorillas. Nature. 2010;467:420–5. 10.1038/nature09442 20864995 PMC2997044

